# Clinical outcomes and causes of arthroscopic hip revision surgery

**DOI:** 10.1038/s41598-018-37708-y

**Published:** 2019-02-04

**Authors:** Guanying Gao, Xin Zhang, Yan Xu, Jianquan Wang

**Affiliations:** Institute of Sports Medicine, Peking University Third Hospital, Peking University Health Science Center, Beijing, China

## Abstract

Hip arthroscopic surgery has become a common technique during the past decade, leading to an increased number of arthroscopic hip revision surgeries. This study aimed to evaluate the clinical outcomes in a series of revision hip arthroscopies to analyse the causes of reoperation in the short to medium-term follow-up. We retrospectively analysed 22 patients who underwent arthroscopic hip revision surgery in our institute. All patients accepted a detailed physical examination and obtained radiographs to measure the centre edge angle, the alpha angle and the offset. Three-dimensional computed tomography was used to evaluate the deformities. The modified Harris Hip Score, visual analogue scale and patient self-reported satisfaction were collected and analysed preoperatively and postoperatively. Our results demonstrated that the modified Harris Hip Score improved from 52.8 to 81.6, and the visual analogue scale decreased from 5.0 to 1.1. Patient self-reported satisfaction was 8.5. In conclusion, patients who underwent arthroscopic hip revision surgery exhibited significant improvement in patient-centred outcomes in the short- to medium-term follow-up, and the outcomes indicated the effectiveness of revision surgery. Residual femoroacetabular impingement and extra-articular impingement are common reasons for arthroscopic hip revision surgery. Misdiagnosis of osteoid osteoma and relapse of synovial chondromatosis are also important reasons for revision.

## Introduction

Over the past decade, hip arthroscopic surgery has developed rapidly, becoming a common technique. According to one meta-analysis involving 6134 mostly adult patients and a second meta-analysis involving 333 patients, the reoperation rates of arthroscopic hip revision surgery were 5.0% and 6.3%, respectively^1,2^. With the increase in arthroscopic hip revision surgery, the clinical outcomes and causes of this revision surgery have become a major current focus. Sardana *et al*.^3^ performed a systematic review of revision hip arthroscopy and concluded that revision hip arthroscopy was a successful treatment to improve functional outcomes and relieve symptoms, and the main common reasons for revision hip arthroscopy included residual femoroacetabular impingement (FAI), labral tears, and chondral lesions. Newman *et al*.^4^ performed a matched-cohort study to compare outcomes in patients who required arthroscopic hip revision surgery with patients undergoing primary hip arthroscopic surgery and concluded that arthroscopic hip revision surgery provided significant improvement in Hip Outcome Score-Activities of Daily Living subscale (HOS-ADL, increased from 65 to 79), modified Harris Hip Score (mHHS, increased from 62 to 79), Western Ontario and McMaster Universities Osteoarthritis Index (WOMAC, improved from 30 to 18), Hip Outcome Score–Sports subscale (HOS-Sports, increased from 40 to 62), and the 12-Item Short Form Health Survey (SF12, increased from 39 to 47). Other studies have proven the effectiveness of arthroscopic hip revision surgery, but studies of reasons for reoperation exhibited different results^5–9^. In addition, all of these studies targeted Caucasian populations. Dudda *et al*.^10^ found that the morphometry of impingement and asphericity were more common in Caucasian compared with Chinese hips in a study of elderly women and Caucasians may be at increased risk of hip osteoarthritis compared with Chinese because of morphologic findings that predispose them to FAI. Chinese people had quite a different lifestyle compared with Caucasians. Morphological differences and different lifestyles may influence the outcomes of revision surgery.

We targeted a population to evaluate the clinical outcomes in a series of revision hip arthroscopies and analysed the causes of reoperation in the short to medium-term follow-up. Evaluating clinical outcomes and causes of revision hip arthroscopies in different ethnic group may reveal new avenues of research.

## Results

### Patient demographics

Twenty-two Chinese non-athlete patients, including male 11 cases and female 11 cases, who underwent arthroscopic hip reversion surgery were included in this study with a minimum of 16-month follow-up. All operations were unilateral. Duration of symptoms after the index surgery was 15.9 months (range from 1 to 36 months). The average follow-up time was 29.5 months (range from 16 to 60 months). The average age of the patients was 32.0 years (range from 15 to 54 years). The average age of the male patients was 31.2 years (range from 16 to 54 years). The average age of the female patients was 30.6 years (range from 15 to 44 years). Average body mass index (BMI) was 22.54 (range from 18.3 to 27.8). Average BMI of females was 21.37 (range from 18.3 to 26.7). Average BMI of males was 24.26 (range from 19.1 to 27.8). Fourteen (63.6%) patients underwent right hip surgeries, and eight (36.3%) underwent left hip surgeries. Four (18.2%) patients experienced persistent pain, and 13 (59.1%) patients experienced relapsed pain after index surgery. Patient demographics are presented in Table 1.

**Table 1:** Demographic Data for Patients Undergoing Arthroscopic Hip Revision Surgery.

Parameter	Data
No. of hips	22
Mean age (range) (yr)	32.0 (15–54)
Mean age of male (range) (yr)	31.2 (16–54)
Mean age of female (range) (yr)	30.6 (15–44)
**Sex (n)**	
Male	11
Female	11
Mean BMI	22.54 (18.3–27.8)
Mean BMI of male	24.26 (19.1–27.8)
Mean BMI of female	21.37 (18.3–26.7)
**Side (n)**	
Left	8
Right	14
Duration of symptoms after the index surgery (mo)	15.9 (1–36)
Mean length of follow-up (range) (mo)	29.5 (16–60)
Persistent pain after index surgery (n) (%)	4 (18.2)
Relapsed pain after index surgery (n) (%)	13 (59.1)

### Physical examination

Provocative physical examination tests are presented in Table 2, including flexion adduction internal rotation (FADIR) test, flexion abduction external rotation (FABER) test, straight leg raise test and iliopsoas impingement. In total, 86.4% of our patients presented with positive FADIR test and FABER test. One presented with positive iliopsoas impingement, and one presented with positive straight leg raise. The average range of motion for the operative hip before surgery is also presented in Table 2. Mean internal rotation was 33.1 ± 10.5° in 0 degree of hip flexion and 31.6 ± 13.6° in 90 degree of hip flexion. Mean external rotation was 30.6 ± 7.9° in 0 degree of hip flexion and 32.5 ± 8.4° in 90 degree of hip flexion. Mean abduction was 40.3 ± 5.9° and mean flexion was 116.9 ± 20.5°.

**Table 2:** Preoperative Physical Examination (Values are presented as the mean ± SD unless otherwise indicated).

	Findings
**Mean internal rotation (degree)**	
0 degree of hip flexion	33.1 ± 10.5
90 degrees of hip flexion	31.6 ± 13.6
**Mean external rotation (degree)**	
0 degree of hip flexion	30.6 ± 7.9
90 degrees of hip flexion	32.5 ± 8.4
Mean abduction (degree)	40.3 ± 5.9
Mean flexion (degree)	116.9 ± 20.5
FADIR (n) (%)	19 (86.4)
FABER (n) (%)	19 (86.4)
Iliopsoas impingement (n) (%)	1 (4.5)
Straight leg raise (n) (%)	1 (4.5)

### Radiological findings

As shown in Table 3, the mean joint space was 4.41 ± 0.70 mm (range from 2.69 mm to 5.78 mm) preoperatively and 4.39 ± 0.67 mm (range from 2.87 mm to 5.79 mm) postoperatively (P = 0.90). The mean anterior centre-edge (ACE) angle was 40.45 ± 7.54° (range from 26.6° to 60.6°) preoperatively and 43.00 ± 6.50° (range from 28.3° to 54.9°) postoperatively (P = 0.14). Mean preoperative alpha angle was 50.52 ± 6.73° (range from 39.7° to 65.6°) with postoperative correction to 41.76 ± 3.56° (range from 36.3° to 48.7°; P = 2.39E-05). Thirteen patients (59.1%) had a preoperative ACE angleå 40°, and 9 patients (40.9%) had a preoperative alpha angleå 50°. Preoperative femoral offset was 7.78 ± 2.17 mm (range from 6.19 mm to 11.11 mm), and postoperative femoral offset was 10.07 ± 2.53 mm (range from 6.08 mm to 15.15 mm) (P = 0.0022). Preoperative crossover sign was identified in 7 (31.8%) patients preoperatively and 0 patients postoperatively.

**Table 3:** Radiological Findings (Values are presented as the mean ± SD unless otherwise indicated).

	Preoperative	Postoperative	P-value
Joint space	4.41 ± 0.70	4.39 ± 0.67	0.9
ACE angle	40.45 ± 7.54	43.00 ± 6.50	0.14
Alpha angle	50.52 ± 6.73	41.76 ± 3.56	2.39E-05
Femoral offset	7.78 ± 2.17	10.07 ± 2.53	0.0022
Crossover sign	7	0	

### Diagnosis and surgical procedure

**Figure 1:**
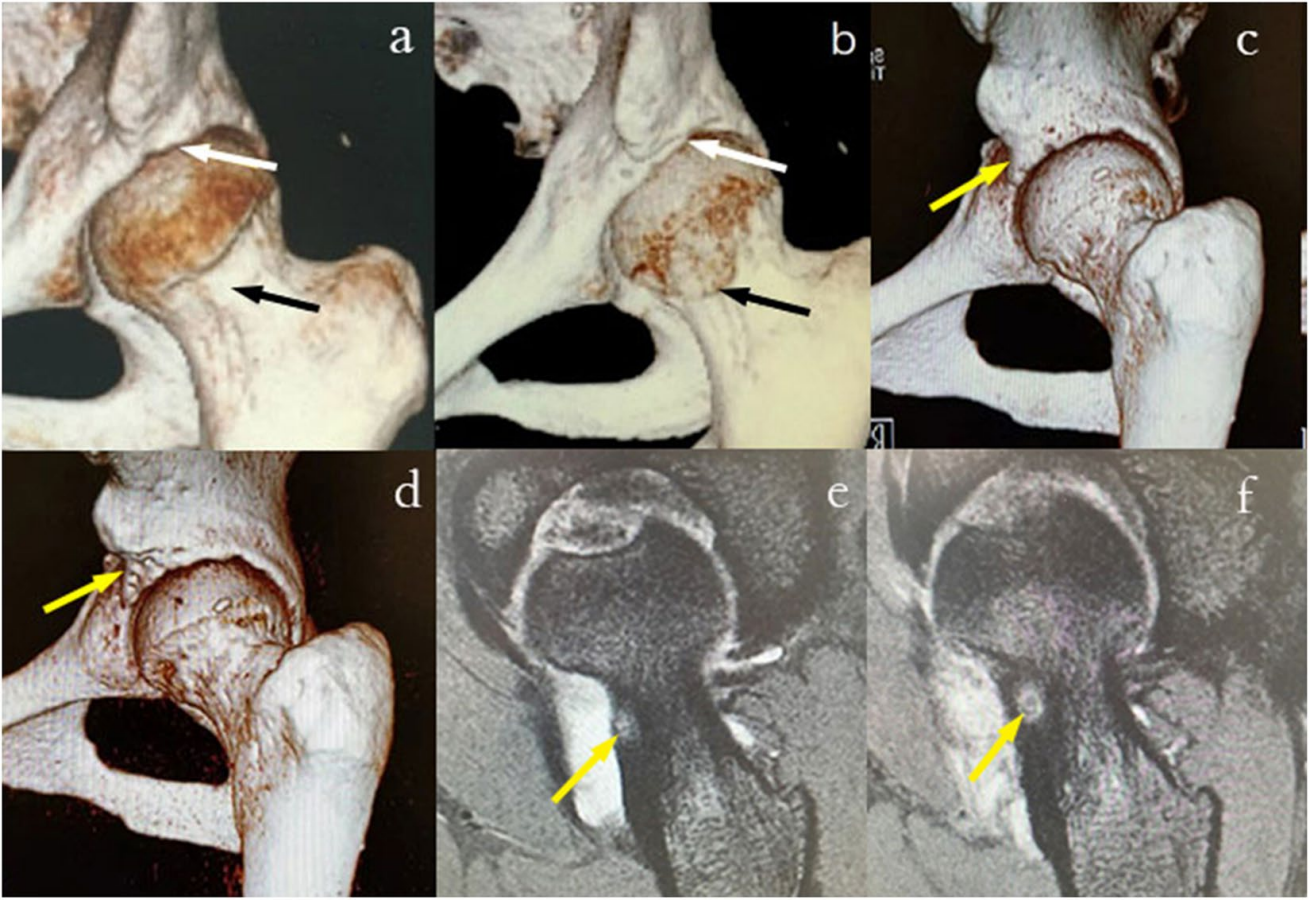
Preoperative and postoperative 3D CT and MRI. (**a**) Preoperative residual cam (black arrow) and pincer deformity (white arrow). (**b**) The outcome of femoral osteoplasty (black arrow) and acetabuloplasty (white arrow) after revision surgery (same patient as presented in (**a**). (**c**) Preoperative anterior inferior iliac spine/subspine impingement (yellow arrow). (**d**) The outcome of focal subspinal decompression (yellow arrow) after revision surgery (same patient as presented in (**c**). (**e**) Misdiagnosis of osteoid osteoma (yellow arrow) before the first arthroscopic surgery. (**f**) The image of osteoid osteoma (yellow arrow) before revision surgery (same patient as presented in (**e**).

Preoperative diagnoses before index surgery and revision surgery are presented in Table 4. Eighteen (81.8%) patients had cam deformity, 12 (54.5%) had pincer deformity, 5 (22.7%) patients had synovial chondromatosis, 17 (77.3%) patients had labral tear and 1 (4.5%) had osteoid osteoma before index surgery (Fig. 1). Eighteen (81.8%) patients had residual cam deformity, 11 (50%) had residual pincer deformity and 3 (13.6%) had extra-articular impingement before revision surgery (Fig. 1). We also found that 16 (72.7%) patients presented with labral tear. In addition, 4 (18.2%) patients had synovial chondromatosis, and 3 (13.6%) had osteoid osteoma. Three patients with osteoid osteoma and 3 patients with extra-articular impingement were misdiagnosed when they underwent the first arthroscopic surgery (Fig. 1). Four in five patients with synovial chondromatosis had a relapse after index surgery. Surgical procedures are presented in Table 5. The majority of patients underwent labral reconstruction (63.6%), femoral osteoplasty (77.3%) and acetabuloplasty (45.5%). Intraoperative cartilage damage findings are presented in Table 6.

**Table 4:** Preoperative Diagnoses Before Index Surgery and Revision Surgery.

Diagnosis	Before Index Surgery, n (%)	Before Revision Surgery, n (%)
Cam deformity	18 (81.8)	18 (81.8)
Pincer deformity	12 (54.5)	11 (50)
Hip joint synovial chondromatosis	5 (22.7)	4 (18.2)
Osteoid osteoma	1 (4.5)	3 (13.6)
Labral tear	17 (77.3)	16 (72.7)
Synovitis	3 (13.6)	1 (4.5)
Stiffness of joint	0 (0)	1 (4.5)
Adhesive capsulitis	0 (0)	1 (4.5)
Iliopsoas impingement	0 (0)	1 (4.5)
Anterior inferior iliac spine/subspine impingement	0 (0)	2 (9.1)
Femoral neck bone cyst	1 (4.5)	1 (4.5)

**Table 5:** Surgical Procedures.

Procedure	n (%)
Capsular release	1 (4.5)
Chondroplasty	1 (4.5)
Labral reconstruction	14 (63.6)
Labral debridement	3 (13.6)
Femoral osteoplasty	17 (77.3)
Acetabuloplasty	10 (45.5)
Iliopsoas release	1 (4.5)
Focal subspinal decompression	2 (9.1)
Loose body removal	2 (9.1)
Synovectomy	2 (9.1)
Excision of osteoid osteoma	3 (13.6)
Synovial chondromatosis debridement	4 (18.2)
Bone cystectomy	1 (4.5)
Microfracture	1 (4.5)

**Table 6:** Intraoperative Cartilage Damage Findings.

Classification (Outerbridge)	n (%)
**Acetabular cartilage damage**	
0	11 (50)
1	0 (0)
2	5 (22.7)
3	4 (18.2)
4	2 (9.1)
**Femoral cartilage damage**	
0	21 (95.5)
1	0 (0)
2	1 (4.5)
3	0 (0)
4	0 (0)

### Patient-centred outcomes

**Figure 2:**
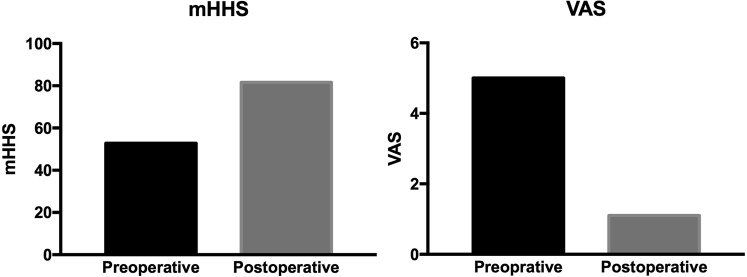
Preoperative and postoperative patient-centred outcomes scores for patients who underwent arthroscopic hip revision surgery.

The modified Harris Hip Score (mHHS) improved from 52.8 (33 to 71) preoperatively to 81.6 (52 to 91) postoperatively, and visual analogue scale (VAS) decreased from 5.0 (3 to 7) to 1.1 (0 to 5) (Fig. 2). No patient required total hip arthroplasty. No complications, such as hip dislocation or fracture, were reported. During the study period, no patient underwent a second revision hip arthroscopy. All results demonstrated statistically significant improvement (P < 0.01). Patient self-reported satisfaction was 8.5 (4 to 10).

## Discussion

The results of this study indicated that arthroscopic hip revision surgery could provide good clinical outcomes for patients who need reoperation. The improvement of mHHS and VAS is statistically significant. Previous report demonstrated that capsulolabral adhesions, failure of labral debridement, and failure to address FAI were the most common reasons precipitating revision hip arthroscopic surgery^11^. In this study, residual FAI accounted for 86.4% of patients who underwent arthroscopic hip revision surgery. Labral and cartilage damage found in the revision surgery corresponded to the site of residual FAI. Gupta *et al*.^12^ evaluated 70 revision hip arthroscopies and found that preoperative diagnoses included 92.9% labral tears with 58.6% residual cam deformity and 51.4% residual pincer deformity. Philippon *et al*.^5^ evaluated 37 revision hip arthroscopies and found that 95% of revision hip arthroscopic surgeries were caused by unaddressed or residual FAI. Domb *et al*.^13^ reported the results of a 2-year follow-up of 47 revisions by hip arthroscopy, and FAI that was unaddressed or inadequately addressed was present at the time of revision in 66% of hips. Gupta *et al*.^12^ reported that 41 (59%) and 36 (51.4%) patients presented with persistent cam and pincer abnormalities before revision surgery. Newman *et al*.^11^ reported 21 (50%) patients had cam and pincer impingement, 9 (21.4%) had cam impingement, 3 (7.1%) had pincer impingement, and 9 (21.4%) had painful adhesions preoperatively in the revision group. Aprato *et al*.^6^ evaluated a total of 63 patients who underwent arthroscopic hip revision surgery and reported that chondral lesion associated with labral re-injury was the most common finding at revision surgery, and only 31% of patients underwent revision for persistent FAI. Underlying residual bone, which was not corrected at the index surgery, is the reason for such a high incidence of labral tears and is also an important reason for reoperation. Compared with the above studies, we find that the main reasons for revision surgery do not differ between Chinese and Caucasian individuals.

In addition to residual FAI, extra-articular hip impingement was also an important reason leading to revision surgery. Extra-articular hip impingement can be classified into iliopsoas impingement, anterior inferior iliac spine/subspine impingement, ischiofemoral impingement, and greater trochanteric/pelvic impingement^14^. To date, extra-articular hip impingement has not been well recognized. In this study, one patient was diagnosed with FAI before the index surgery and experienced persistent hip pain after surgery. Iliopsoas impingement was identified before revision surgery of this patient, and symptoms were relieved after iliopsoas release. Two cases of anterior inferior iliac spine/subspine impingement were identified in this study, and symptoms were also relieved after arthroscopic focal subspinal decompression. Ricciardi *et al*.^7^ evaluated 147 patients who underwent revision hip preservation surgery and found that residual or unaddressed intra- and extra-articular impingement were the most common causes.

It is important to note that misdiagnosis of osteoid osteoma is also a reason easily overlooked for revision surgery. Osteoid osteoma of the hip is a relatively rare diagnosis, which can closely mimic symptomatic presentation of FAI, and arthroscopic surgery can be especially effective in patients with concomitant FAI^15^. Osteoid osteoma can be diagnosed by MRI. Osteoid osteoma should be carefully excluded before surgery in case of misdiagnosis and revision surgery. Synovial chondromatosis is also a relatively rare disease involving cartilaginous metaplasia of synovial tissue, and treatment via synovectomy and loose body removal results in high revision rates^16^. Given the high recurrence rate, synovial chondromatosis is also an important reason for revision surgery.

In this study, the mHHS improved from 52.8 preoperatively to 81.6 postoperatively, and VAS improved from 5.0 to 1.1. Domb *et al*.^13^ reported that mHHS improved from 55 to 72 and VAS improved from 7.3 to 3.9. Larson *et al*.^9^ reported that mHHS improved from 62.1 to 79.9 and VAS improved from 4.4 to 3.0. Philippon *et al*.^5^ reported that mHHS improved from an average of 56 to 77 after one-year follow-up. Aprato *et al*.^6^ reported that mHHS improved from 54 to 59 after 3-year follow-up. Ricciardi *et al*.^7^ reported that mHHS improved from 56.6 to 72.1 after 15-month follow-up. Gupta *et al*.^12^ reported that mHHS improved from 57.8 to 73.7 after 2-year follow-up. Newman *et al*.^11^ reported that mHHS improved from 55.3 to 74.3 after a minimum 2-year follow-up. Only one study did not achieve postoperative mHHS equal to the other studies. Aprato *et al*.^6^ reported that the presence of chondral lesions was associated with reduced postoperative mHHS. It is notable that one patient did not fare as well as others with mHHS (52) and VAS (5) after revision surgery. This patient had the lowest mHHS (33) and the highest VAS (7) and the severest joint degeneration before revision surgery. This finding suggests that we might need to choose patients strictly, and patients with low mHHS, high VAS and severe joint degeneration may have poor operative effect after revision surgery. Compared with the above studies, we believe that the outcomes of revision surgery reveal no differences between Chinese and Caucasian individuals. Given the lack of a similar study among Chinese or Asians, further studies are needed.

Preoperative 3-dimensional computed tomography (3D CT) is helpful for identifying pathological areas preoperatively and especially for residual FAI and extra-articular impingement^12^. Here, 3D CT can compensate for the limitation of radiography and the limited vision of arthroscopy to increase accuracy and success rate of surgery and decrease reoperation^17,18^. In addition, 3D CT can improve diagnostic assessment of patients with suspected FAI or residual FAI and is recommended uncertainty is noted^12,19^. We used 3D CT to help identify pathological areas and demonstrate the amount of bony osteoplasty.

In conclusion, patients who underwent arthroscopic hip revision surgery exhibited significant improvement in patient-centred outcomes in the short to medium-term follow-up, and the outcomes indicated the effectiveness of revision surgery. Residual FAI and extra-articular impingement are common reasons for arthroscopic hip revision surgery. In addition, misdiagnosis of osteoid osteoma and relapse of synovial chondromatosis are also important reasons for revision surgery.

## Method

### Patients

Twenty-two consecutive patients who underwent arthroscopic hip revision surgery in our institute from July 2012 to July 2016 were included in this study. The subjects were all Chinese and not athletes. All patients were undergoing revision surgery for the first time. The Ethics Committee of the Third Hospital of Peking University approved this study. All participants signed informed consent under the Ethics Committee of the Third Hospital of Peking University approved protocol. All methods were performed in accordance with the guidelines and regulations of the Ethics Committee of the Third Hospital of Peking University.

The inclusion criteria were as follows:(1) the index surgery was arthroscopic hip surgery; (2) hip joint space >2 mm in the affected side and Tönnis grade was I or II; (3) persistent hip pain and limited activity that was aggravated or aggravated after remission with pain mainly in the frontal groin area, and conservative treatment was ineffective; or (4) an obvious cause of their pain was discovered. The following exclusion criteria were employed: (1) acetabular fracture, (2) avascular necrosis, (3) Legg-Calve-Perthes disease, (4) Ehlers-Danlos syndrome, and (5) pigmented villonodular synovitis.

### Surgical procedure

Arthroscopic hip reversion surgery was performed in the supine position with a standard fracture table and custom perineal post. Traction was applied to the operative extremity to ensure that the operative side hip joint space was 8 to 10 mm under fluoroscopic guidance. In addition, the hip joint was fully adducted, and internal rotation was performed. According to Philippon and Sehenker’s method, a spinal needle was inserted under fluoroscopic guidance to establish the anterolateral portal. Then, the anterior portal was made under direct visualization by viewing from the anterolateral portal^20^. Arthroscopic knife or radio frequency ablation device was used to complete capsulotomy, and a detailed inspection of the central compartment was performed to assess the acetabular rim, acetabular labrum, articular cartilage and ligamentum teres. Labral repair or labral debridement was performed according to the injury. We tried to suture the damaged acetabular labrum instead of resection because over-resecting the acetabular labrum will destabilize the hip joint. Depending on the size, location, and Outerbridge classification system, cartilage damages were treated with arthroscopic debridement for partial-thickness lesions or arthroscopic microfracture for full-thickness lesions^21^. If a cam bump in the head-neck junction or acetabular overcoverage was identified, femoral osteoplasty or acetabuloplasty was performed. Iliopsoas release was performed in patients with a positive iliopsoas impingement test result. Debride trochanteric bursa and footprint of gluteus medius muscles was performed in patients who had trochanteric tenderness or inflammation in the footprint of gluteus medius muscles as demonstrated by MRI before surgery. Focal subspinal decompression, loose body removal, synovectomy, excision of osteoid osteoma, synovial chondromatosis debridement and bone cystectomy were performed when necessary.

### Postoperative protocol

Begin Ankle pump, quadriceps strengthening and other isometric exercises were initiated 1 or 2 days after surgery. Hip passive range of motion (ROM) exercise as tolerated was initiated 3 or 4 days after surgery. Partial weight bearing with crutches was initiated 3 to 7 days after surgery, and passive ROM exercise and active ROM exercises were performed as tolerated after 4 weeks postoperatively. Patients should advance to full weight bearing by 6 weeks and restore symmetrical hip ROM 6 weeks after surgery. Patients can begin jogging and advance to running 3 to 6 months after surgery.

### Outcome measures

A detailed physical examination was conducted on all hips before surgery during a routine physical exam without anaesthesia. A half-circle bodied metal goniometer with a moveable arm and scale marked in 5° increments was used^22^. Internal and external rotation were measured while the patient was in the supine position with both the hip and knee flexed at 90° or 0°^22,23^. Abduction and flexion were also recorded^22^. FADIR or FABER tests are considered positive if hip or groin pain was elicited when the hip was placed in 90° of flexion and then adduction and internal rotation or flexion, abduction and external rotation applied^23^. Supine anteroposterior (AP) pelvis, cross-table lateral radiographs and Dunn view (45 degrees) radiographs were obtained on all patients to measure the ACE angle^24,25^, the alpha angle^26,27^, the joint space, and the degree of femoral head-neck offset. The ACE angle was measured from radiographs using the method described by Ömeroglu and Wiberg *et al*.^28^. The alpha angle was measured from radiographs using the method described by Barton and Notzli *et al*.^26,27^. The joint space was measured from radiographs using the method described by Reis *et al*.^29^. The offset was evaluated on cross-table lateral radiographs using the method described by Notzli *et al*.^27^. Three-dimensional computed tomography (3D CT) was used to evaluate the deformities and provide intuitive view. One observer performed all measurements. The mHHS, VAS and patient self-reported satisfaction with surgical outcomes (on a scale of 0 to 10 with 10 being the best) were collected and analysed retrospectively.

### Statistical analysis

The 2-tailed paired t-test was used to evaluate significance between preoperative and postoperative groups. P-values of <0.05 were considered statistically significant. Statistical analysis was performed using the SPSS software (version 11; SPSS Inc.).

## Data Availability

The datasets generated during and/or analysed during the current study are available from the corresponding author on reasonable request.
